# Intratumor injection of BCG Ag85A high-affinity peptides enhanced anti-tumor efficacy in PPD-positive melanoma

**DOI:** 10.1007/s00262-024-03693-7

**Published:** 2024-04-17

**Authors:** Lanqun Qin, Guiying Zhang, Yirong Wu, Yueling Yang, Zhengyun Zou

**Affiliations:** 1 Department of the Comprehensive Cancer Center, Nanjing Drum Tower Hospital, Clinical College of Nanjing Medical University, 321 Zhongshan Road, Nanjing, 210008 China; 2 Department of the Comprehensive Cancer Center, Nanjing Drum Tower Hospital, The Affiliated Hospital of Nanjing University Medical School, Nanjing, China; 3 Department of the Comprehensive Cancer Center, Nanjing Drum Tower Hospital, Clinical College of Traditional Chinese and Western Medicine, Nanjing University of Chinese Medicine, Nanjing, China

**Keywords:** BCG, Ag85A, Antigen delivery, MHC-peptide complex, PD-L1

## Abstract

As one of the scheduled immunization vaccines worldwide, virtually all individuals have been vaccinated with BCG vaccine. In order to verify the hypothesis that delivering BCG high-affinity peptides to tumor areas could activate the existing BCG memory T cells to attack tumor, we firstly predicted the HLA-A*0201 high-affinity peptides of BCG Ag85A protein (KLIANNTRV, GLPVEYLQV), and then, A375 melanoma cells and HLA-A*0201 PBMCs (from PPD-positive adults) were added to co-incubated with the predicted peptides in vitro. We found that the predicted BCG high-affinity peptides could be directly loaded onto the surface of tumor cells, enhancing the tumor-killing efficacy of PBMCs from PPD-positive volunteer. Then, we constructed PPD-positive mice model bearing B16F10 subcutaneous tumors and found that intratumor injection of BCG Ag85A high-affinity peptides (SGGANSPAL, YHPQQFVYAGAMSGLLD) enhanced the anti-tumor efficacy in PPD-positive melanoma mice. Along with the better anti-tumor efficacy, the expression of PDL1 on tumor cell surface was also increased, and stronger antitumor effects occurred when further combined with anti-PD1 antibody. For microenvironment analysis, the proportion of effector memory T cells was increased and the better treatment efficacy may be attributed to the elevated effector memory CD4 + T cells within the tumor. In conclusion, using the existing immune response of BCG vaccine by delivering high-affinity peptides of BCG to tumor area is a safe and promising therapy for cancer.

## Introduction

The tumor microenvironment (TME), commonly regarded as an active facilitator of cancer progression, is a complex microecosystem consisting of tumor cells, immune cells, and non-cellular components such as the extracellular matrix and cytokines [2, 40]. As the important and vanguard cells responsible for tumor attack, only a small number of tumor-infiltrating T cells exhibit tumor specificity, while the majority are T cells not capable of recognizing antigens related to tumors. Despite their presence within tumors, these T cells fail to attack tumor cells if tumor cells do not express the antigens that they can recognize and are thus referred to as bystander T cells [13, 24, 25].

Studies have demonstrated the presence of virus bystander T cells in colorectal cancer, lung cancer, and melanoma [9, 37]. The presence of virus-specific T cells in the peripheral blood could 100% predict their presence in the paired tumor tissues [34]. Meanwhile, intratumoral peptide injection enhances the antigenicity of tumor cells, regardless of whether the tumor cells originally expressed the antigen, enabling the recognition and attack of tumor cells by their corresponding bystander T cells [32]. For instance, antibody-peptide epitope conjugates carrying high-affinity peptide NLVPMVATV of Cytomegalovirus (CMV) or GLCTLVAML of Epstein-Barr Virus (EBV) could specifically activate virus-reactive effector T cell in the TME [26]. Meanwhile, antiviral T-cell activation induced activation of both the innate and adaptive immune system within the tumor and could synergize with checkpoint blockade inhibitors to eliminate normally resistant tumors [34]. However, similar attempts have not yet been made in bacteria, which are present in nearly all tumors [31]. More importantly, bacterial-derived peptides exhibit significantly greater hydrophobicity, a property that renders these peptides more suitable for antigen presentation and antigen recognition by T cells [5, 18, 42].

As an intracellular bacterium, tuberculosis (TB) predominantly activates cellular immunity and is commonly prevented through Bacillus Calmette–Guerin (BCG) vaccination [10]. As one of the scheduled immunization vaccines worldwide, virtually all individuals have been vaccinated with BCG, resulting in a prevalent memory immune response to BCG in the human body. Nowadays, BCG is widely used to in clinical. BCG intravesical perfusion is considered the standard adjuvant immunotherapy for the treatment of Nonmuscle-Invasive Bladder Cancer (NMIBC) [21]. Mechanically speaking, in addition to the recognized non-specific anti-tumor immune response, BCG also promoted the formation of tumor-specific immune memory and the proliferation of tumor antigen-specific T cells [16]. However, some peptides of BCG, after being swallowed and digested by antigen presentation cells (APCs), could be presented by major histocompatibility complex (MHC) molecules to activate BCG-specific T cells, and some peptides could load onto tumor cell surfaces as well. It is possible that when BCG activated tumor antigen-specific T cells, it may also activate BCG antigen-specific T cells to attack tumors, showing a bystander effect. No such speculation has been suggested in the literature to date.

Ag85A protein, a protein common to mycobacterium tuberculosis (TB) and BCG, is the main protective protein that induces protective immunity [19, 45].Here, we postulated and verified that high-affinity peptides of Ag85A protein can be loaded onto tumor cells via intratumor injection so that tumor cells that did not express these peptides previously can now be recognized and then targeted attacked by intratumoral existing BCG antigen-specific bystander T cells.

## Results

### PPD test reflected the memory immune response in vivo.

**Fig. 1:**
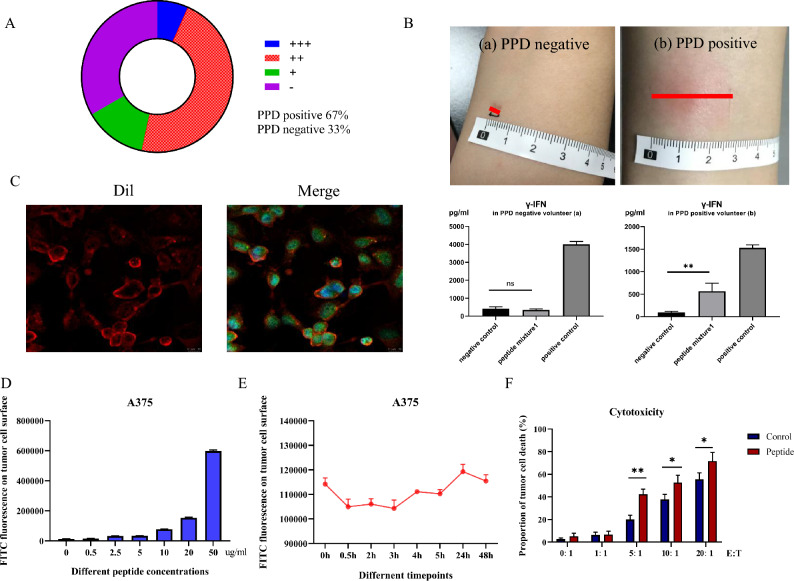
Peptides could be stably loaded onto the tumor cell membrane and enhanced the killing efficiency of T cells for PPD-positive volunteers. **A** PPD test in 15 healthy volunteers. **B** *γ*-IFN secretion of PBMCs in PPD negative volunteer a and PPD-positive volunteer b after co-incubation with the predicted peptide mixture1. **C** Confocal images of GLPVEYLQV-FITC (green) co-incubated with A375 melanoma cells (HLA-A*02:01). The cell nuclei were stained with DAPI dye (blue) and cell membranes were stained with Dil dye (red). **D** Fluorescence on tumor cell s when co-incubated with different peptides concentrations (0, 2.5, 5, 10, 20, 50 µg/mL). **E** Fluorescence on tumor cell surface when co-incubated with predicted high-affinity peptides (10 µg/mL) at different time points (0, 0.5, 2, 3, 4, 5, 24, 48 h). **F** Killing efficiency of T cells of PPD-positive volunteer with or without BCG high-affinity peptides by CFSE/PI staining. **E** T, effector–target ratio. Scale bar, 100 µm. Differences between groups were analyzed by Student's *t* test or one-way ANOVA test. Data represent mean ± s.e.m.; *n* = 3; ns, not significant; *p < 0.05; **p < 0.01; ***p < 0.001

15 healthy adult volunteers were recruited for PPD test with a positive rate of approximately 66.7% (10/15) (Fig. 1a). Subsequently, PBMCs from HLA-A*0201 volunteers were co-incubated with the predicted BCG Ag85A high-affinity peptide mixture1 for 24 h. It was observed that *γ*-IFN secretion increased significantly in PPD-positive volunteer B, while there was no change in PPD negative volunteer A (Fig. 1b), indicating that the PPD test can reflect the intensity of BCG memory immune response in vivo.

### Peptides were stably loaded onto the tumor cell membrane.

Human melanoma cell line A375 cells (HLA-A*0201) were co-incubated with BCG high-affinity peptide GLPVEYLQV-FITC (with green fluorescence). The cell nuclei were stained with DAPI dye (with blue fluorescence), and cell membranes were stained with Dil dye (with red fluorescence). Images were captured using a confocal laser microscopy. After merging, the fluorescence of cell membrane turned red into orange, suggesting the successful loading of peptides onto tumor cell. Additionally, significant green fluorescence appeared in the cytoplasm, indicating phagocytosis of peptides by tumor cells (Fig. 1c). Next, the fluorescence intensity on tumor cell surface was detected by flow cytometry. As the concentration of high affinity peptides increased, the fluorescence intensity was also growing, prompting again that peptides can be loaded onto the surface of tumor cells (Fig. 1d). At the same time, there was a large amount of empty HLA molecules on the tumor cell surface, so that the fluorescence intensity did not saturate at high peptide concentrations. Then, fluorescence was detected at different time points and was basically stable 48 h later (Fig. 1e), indicating that peptides can be stably loaded onto the tumor cell membrane.

### BCG high-affinity peptides increased the killing efficiency of PPD-positive PBMCs.

The peptide mixture1 and A375 cells were co-incubated with PBMCs of PPD-positive volunteer (HLA-A*0201) for 24 h. The killing efficiency of PBMCs was assessed using CFSE/PI assay. As the effector-to-target ratio increased, the tumor-killing efficiency of PBMCs also increased, and the addition of peptides further enhanced the killing efficiency (Fig. 1f). Prompting that the predicted BCG high-affinity peptides can enhance the targeted killing efficiency of the memory T cells functionally.

### Construction and verification of BCG immunized mice.

**Fig. 2:**
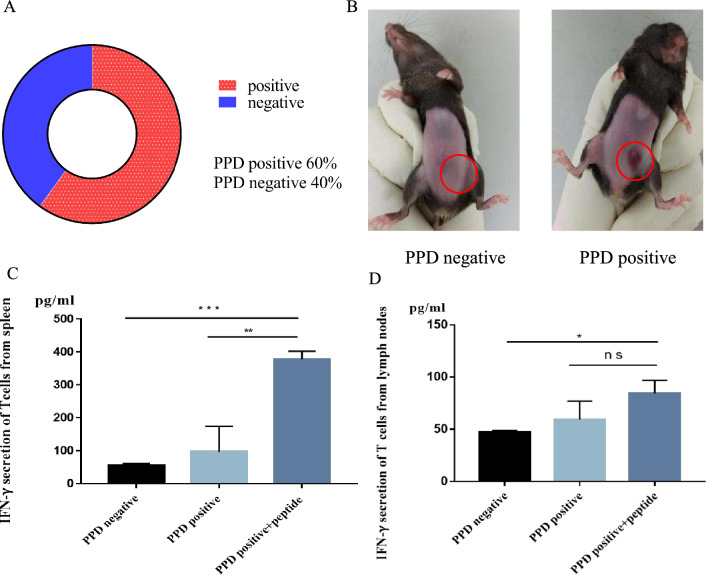
The construction and verification of BCG immunized mice. **A** PPD test for 40 mice immunized with BCG at Day0. **B** Typical images of PPD-positive and PPD negative mice based on the level of local redness or induration. **C**, **D** *γ*-IFN expression in supernatant when T cells from PPD negative mice, PPD-positive mice are cultured in vitro, or T cells from PPD-positive mice co-incubated with BCG high-affinity peptide mixture2 in vitro for 18–24 h. **C** T cells from spleen. **D** T cells from lymph nodes. Data represent mean ± s.e.m.; *n* = 3. one-way ANOVA test. ns, not significant; *p < 0.05; **p < 0.01; ***p < 0.001

A total of 40 PPD negative C57BL/6J mice were immunized with intradermal injections of 0.1 mL of BCG suspension at -Day28 and -Day21, respectively. PPD test was performed again at D0 and 60% (24/60) of mice turned to PPD-positive (Fig. 2a, b). BMDC cells were isolated from the femur and humerus of mice and then cultured into mature DC cells. These cells were co-incubated with T cells from spleen or lymph nodes, followed by the addition of peptide mixture2, and subsequent detection of *γ*-IFN expression in the supernatant. For PPD-positive mice, *γ*-IFN secretion of T cells from both spleen and lymph nodes increased significantly when adding BCG high-affinity peptides (*p* < 0.001 for spleen; *p* < 0.05 for lymph nodes) (Fig. 2c, d). These findings suggest that BCG preimmunization induces a BCG memory immune response in mice, and BCG high-affinity peptides can enhance the tumor-killing efficacy of BCG memory T cells.

### Intratumor injection of Ag85A high-affinity peptides enhanced anti-tumor efficacy in PPD-positive melanoma mice.

**Fig. 3:**
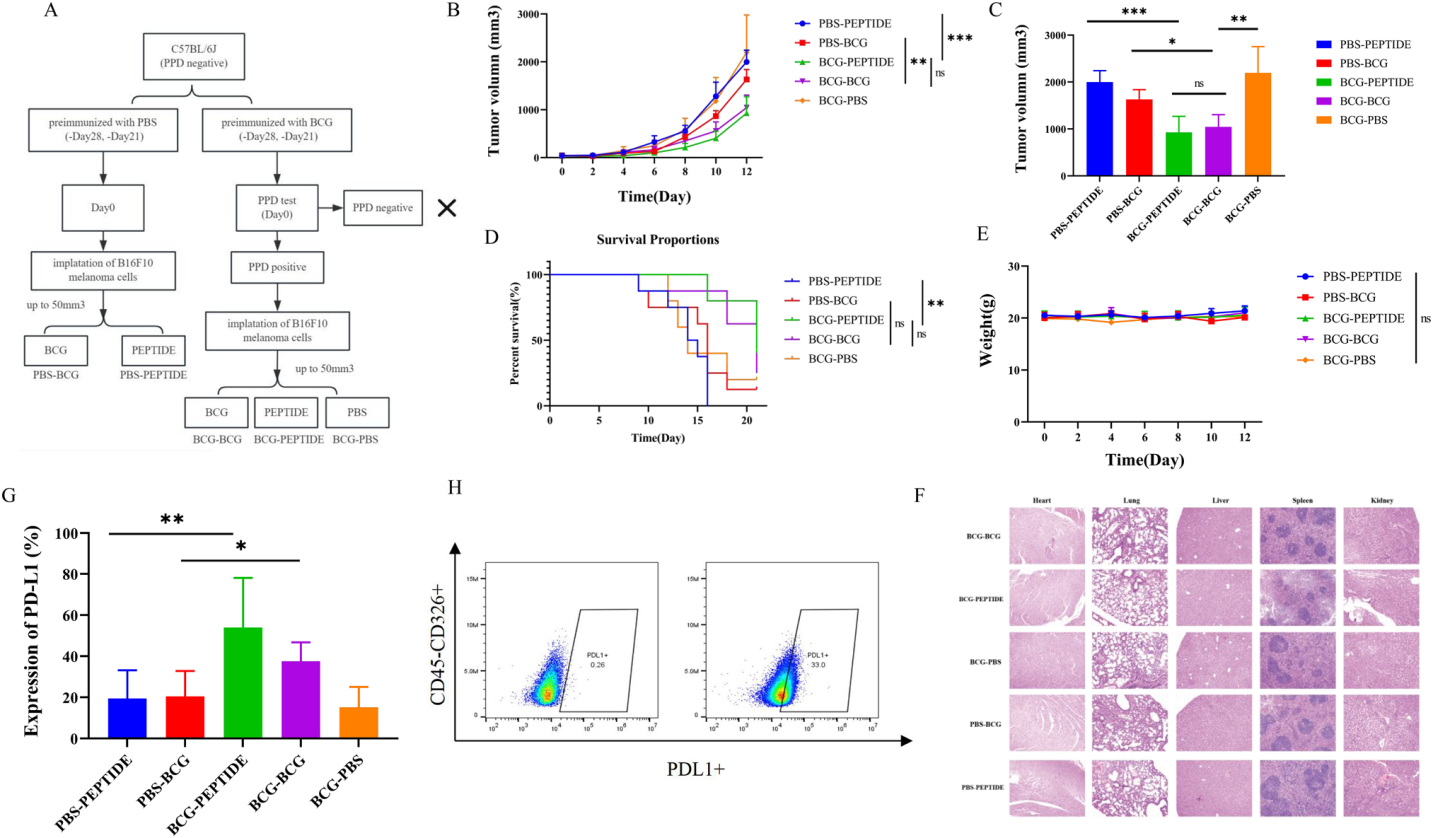
Intratumor injection of BCG Ag85A high-affinity peptides enhanced anti-tumor efficacy in PPD-positive melanoma mice. **A** Study design and grouping (*n* = 6). **B**–**D** Tumor growth profiles (**B**), tumor volume (**C**) and the survival curve of mice (**D**) in different treatment groups. **E** Mice weights of different treatment groups. **F** HE staining of important organs including lungs, livers, spleens, kidneys, and hearts of different treatment groups. **G** Expression of PD-L1 on tumor cells in different treatment groups. **H** Typical images of low and high expression of PD-L1 on tumor cells. Survival curves were analyzed with log-rank test. Tumor volume was analyzed with one-way ANOVA test. Data are represented as mean ± s.e.m., *n* = 6; ns, not significant; *p < 0.05; **p < 0.01; ***p < 0.001

PBS, BCG, or peptide mixture2 were injected intratumorally every 5 days to examine their anti-tumor efficacy (Fig. 3a). Both the BCG-BCG and BCG-PEPTIDE groups showed slower tumor growth and longer survival compared to the non-preimmunized group, and parallel antitumor effects were observed between the BCG-BCG and BCG-PEPTIDE groups (Fig. 3b-d). For mice without prior immunization with BCG, intratumoral injection of BCG (PBS-BCG) had a marginal effect and intratumoral injection of PEPTIDE (PBS-PEPTIDE) showed no therapeutic effect. These findings suggest that BCG high-affinity peptide lacks therapeutic efficacy in the absence of BCG memory T cells. For side effects, no weight loss or acute organ damage was observed during the treatment (Fig. 3e, f). In the end, tumor tissues were collected for microenvironment analysis which revealed a significant increase in PD-L1 expression on tumor cells in both BCG-PEPTIDE group and BCG-BCG group, particularly pronounced in the former group (Fig. 3g, h). Together, the better the therapeutic effect was, the higher the expression of PD-L1 was.

### BCG high-affinity peptide combined with anti-PD1 antibody showed stronger antitumor effect in PPD-positive melanoma mice.

**Fig. 4:**
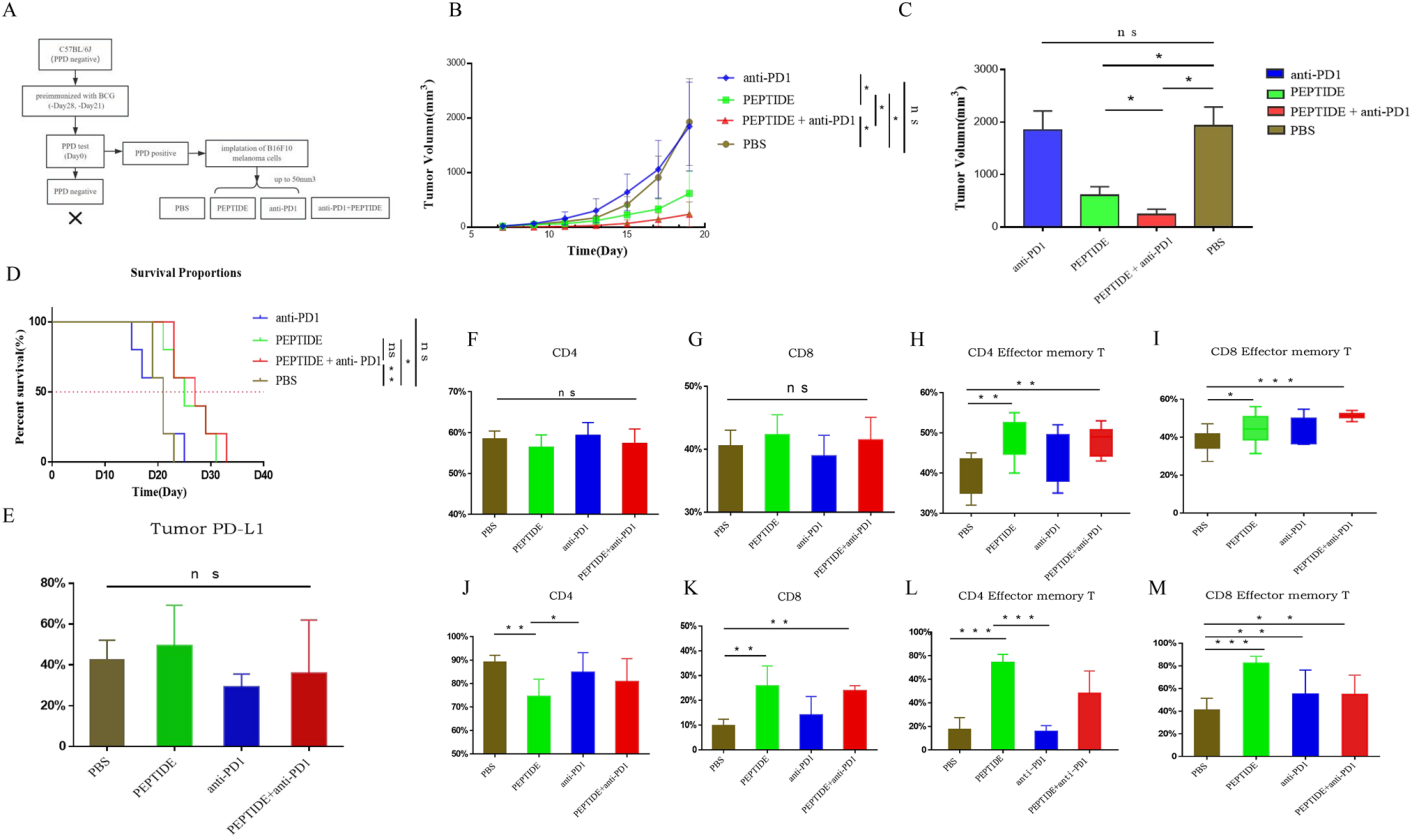
BCG high-affinity peptide injected intratumorally combined with anti-PD1 antibody showed stronger antitumor effect. **A** Study design and grouping (*n* = 5). **B**–**D** Tumor growth profiles (**B**), tumor volume (**C**) and survival curve of mice (**D**) of different treatment groups. **E** Expression of PD-L1 on tumor cells of different treatment groups after treatment. **F–G** The proportion of CD4 + T and CD8 + T cells in lymph nodes. **H–I** The proportion of effector memory CD4 + T and CD8 + T cells in lymph nodes. **J–K** The proportion of CD4 + T and CD8 + T cells within tumor tissues. **L–M** The proportion of effector memory CD4 + T and CD8 + T cells within tumor tissues. Survival curves were analyzed with log-rank test. Tumor volume was analyzed with one-way ANOVA test. Data are represented as mean ± s.e.m., *n* = 5; ns, not significant; *p < 0.05; **p < 0.01; ***p < 0.001

To validate the above observations, we established a BCG-immunized PPD-positive mice model and assessed the combined therapeutic effect with murine anti-PD1 antibody (Fig. 4a). Consistently, mice treated with PEPTIDE + anti-PD1 exhibited significantly slower tumor growth and longer survival compared to those treated only with PEPTIDE (*p* = 0.047). There was no significant difference between PBS and anti-PD1 group as well (Fig. 4b-d).

### Effector memory CD4 + T cells in the tumor microenvironment might be beneficial for better efficacy

To verify the possible causes of the above results from the perspective of immune microenvironment, T cells from the tumor and lymph nodes were separated and prepared as single-cell suspensions for flow cytometry. As the results showed, the proportion of CD4 + T and CD8 + T cells did not change in lymph nodes (Fig. 4f-g), while the proportion of CD8 + T cells within the tumor both increased in the PEPTIDE and PEPTIDE + anti-PD1 group (Fig. 4j-k). Effector memory CD4 + T and CD8 + T cells both increased significantly in lymph nodes and tumors, which may be attributed to the better efficacy of PEPTIDE and PEPTIDE + anti-PD1 groups (Fig. 4h-i, l-m). It is suggested that injecting BCG high-affinity peptide intratumorally can activate BCG memory T cells within the tumor after immunization. Notably, the anti-PD1 group showed similar efficacy to the PBS group. Although anti-PD1 group also showed an increase in effector memory CD8 + T cells, effector memory CD4 + T cells were similar to those in the PBS group. We speculated that intratumoral effector memory CD4 + T cells may play a more important role in this process. Finally, we re-examined the expression of PD-L1 on tumor cells. The results showed that peptide injected intratumorally could increase the expression of PD-L1 on tumor cells, but could be decreased by combining with anti-PD1 antibody (Fig. 4e).

## Discussion

As a wildly utilized immune adjuvant, dating back to 1974, intratumoral injection of BCG resulted in the regression of 90% of injected melanoma lesions and 17% of non-injected lesions [29]. BCG is also currently the standard treatment for stage III melanoma [4]. Whereas, repeated intratumoral administration of BCG has also been associated with a number of systemic side effects, such as fever, local lymphadenitis, and sporadic systemic infections, particularly in immunocompromised cancer patients [14, 39]. Therefore, our focus lies on how to maximize the anti-tumor efficacy of BCG while ensuring safety. Our study validated again that intratumoral injection of BCG high-affinity peptides enhances tumor cell antigenicity [32]. Once these high-affinity peptides are delivered into the tumor microenvironment, they may be directly loaded onto the empty MHC molecules on the surface of tumor cells to form the peptide-MHC complexes according to the speculation of Millar DG et al. It was not clear whether antigen presenting cells, such as dendritic cells, were involved in the whole process. However, broadly speaking, tumor cells also belong to the category of APCs. Once the peptides were loaded onto the tumors, they promptly activate BCG antigen-specific memory T cells to initiate an innate and adaptive immune response to attack those peptide-loaded tumor cells.

Firstly, most adults have a memory immune response to BCG and the BCG high-affinity peptides can be efficiently loaded onto the surface of tumor cells. Here, volunteers with positive PPD could not be ruled out of TB infection. But the premise of our research was to obtain memory immunity against mycobacterium, either through BCG vaccination or infection with TB. Since Ag85A is an immunogenic protein shared by TB and BCG, the high-affinity peptides we predicted for Ag85A are also applicable to both. In a word, this anti-tumor strategy is also suitable for tumor patients infected with TB. Secondly, BCG high-affinity peptides can activate preexisting BCG memory T cells and enhance their anti-tumor cytotoxicity when co-cultured with PBMCs, though most of the effect is nonspecific from the added peptide. This is because PBMCs containing T cells, APCs, NK cells and macrophages and so on. Innate immune cells, including NK cells and macrophages, could directly kill tumor cells. The addition of BCG high-affinity peptides to tumors only activated the existing memory T cells to BCG, making these bystander T cells exerting anti-tumor effects.

Subsequently, we pre-immunized mice to mimic the state of human BCG vaccination and observed that the PPD-positive mice exhibited better treatment outcomes than those not immunized with BCG, indicating the activation of stored BCG memory immune response within the body. However, there was no significant difference between the BCG-BCG group and the BCG-PEPTIDE group. Further explorations may be required to determine optimal peptide dosages or to design improved peptide combinations. But the phenomenon at least suggested that high-affinity peptides could replace BCG while maintaining equivalent therapeutic efficacy. For mice without prior immunization with BCG, intratumoral injection of PEPTIDE (PBS-PEPTIDE) showed no therapeutic effect. Verified from the side once again that the BCG high-affinity peptide could not work without the existing BCG memory T cells. Finally, higher therapeutic efficacy was associated with elevated PD-L1 expression levels, consisting with previous research [11, 23, 35] as T-cell activation upregulates PD-1 expression leading to increased PD-L1 expression on tumor cells due to immune evasion [43].

Thus, for those PPD-positive melanoma mice, by injecting BCG Ag85A high-affinity peptides into tumors, antitumor efficiency could be improved due to the followed two reasons: One for the BCG high-affinity peptides could be loaded onto the tumor cell membrane, and the other for there were enough BCG memory bystander T cells in the tumor microenvironment.

Effector memory T cells in the TME, in particular effector memory CD4 + T cells, may play a crucial role in this process. This aligns with the observation that both natural TB infection and BCG vaccination induce comparable protective immunity mechanisms that rely primarily on CD4 + T cells, along with some contribution from CD8 + T cells [22, 27]. Further expand the scope, bacterial immunotherapy for cancer also induces CD4-dependent tumor-specific immunity through tumor-intrinsic *γ*-IFN signaling [3]. This is why anti-PD1 group showed increased proportion of effector memory CD8 + T cells in our study but failed to exhibit any therapeutic effect. Certainly, we have to acknowledge that there were other cells, cytokines, and gene pathways at work in TME that we have not yet detected. More importantly, polyfunctional CD4 T cells were the predominant T cell population 2 and 8 months after BCG vaccination, but were unable to detect these cells at 14 months [7]. The optimal treatment time period after BCG immunization also needs to be further explored.

A prerequisite for T cells to eliminate tumor cells is the recognition of the peptide-MHC complex on the surface of tumor cells by T cell receptors (TCRs) [33]. However, due to tumor cell heterogeneity, not all cells express the same antigen. Additionally, immune escape can occur through antigen modulation and covering, leading to loss of antigen expression on tumor cell surfaces and limiting the efficacy of T cell immunotherapy [8, 41, 44]. To enhance tumor antigenicity, intratumoral injection of high-affinity peptides can be employed, which has been shown to augment anti-tumor activity and induce a systemic immune response compared to conventional subcutaneous injection [17]. It should be noted that this study was limited to a mouse model of melanoma as our therapeutic strategy holds promise for universal application in cancer treatment. Considering that not all tumors have injectable lesions on their surface, various delivery systems have been investigated, such as copolymer-mediated epitope delivery systems [26, 46] and nanoliposomes-mediated delivery systems [15, 20, 30]. Meanwhile, it is difficult to spread the peptides over the whole tumor by intratumoral injection, especially against large tumors. Multipoint intratumoral injection may ameliorate this dilemma.

In recent years, studies have demonstrated that intravenous administration of BCG in mice can enhance NK and T cell-mediated anti-tumor immunity. Higher doses of intravenously injected BCG may elevate the levels of protective T cells in the lungs, thereby significantly enhancing its efficacy against TB infection [6, 28]. Although this could potentially be a future direction for BCG immunization, it is crucial to consider the safety concerns associated with cancer patients who have compromised immune function.

Expanding our perspective, we propose that since COVID-19 has become a global pandemic since 2020, there may be shared immunity to COVID-19 within the human body. Similarly, delivering high-affinity antigens derived from COVID-19 into tumors could be an alternative approach for anti-tumor purposes. Previous attempts have also been made to exploit COVID-19 against tumors. For instance, the SARS-CoV-2 S1 protein has shown potential in inducing apoptosis in lung cancer cells [36]. However, it should be noted that these findings are preliminary explorations and require clinical validation. Nevertheless, they provide evidence supporting the possibility of leveraging existing pathogen-specific immunity for anti-tumor strategies and substituting pathogens with high-affinity peptides. While the large number of virus types and rapid mutation, of course, is a major obstacle to clinical conversion.

In short, this is a broad-spectrum cancer treatment strategy with good anti-cancer effects and fewer side effects that can be easily converted to the clinic. More basic and clinical studies are needed in future.

## Methods and materials

### PPD test

Healthy adult volunteers were recruited for intradermal injection of 0.1mL Purified Protein Derivative of Tuberculin (TB-PPD) (Beijing Sanroad Biological Products Co., Ltd., China) from the middle flexion of the left arm. The results were interpreted 72 h after injection as: (1) " + ": redness, induration diameter of 5-9mm. (2) " +  + ": redness, induration diameter of 10-19mm. (3) " +  +  + ": redness, induration diameter greater than 20mm. (4) " +  +  +  + ": local symptoms such as redness and swelling, as well as systemic symptoms such as herpes, necrosis and fever. (5) "-": redness, induration diameter less than 5 mm.

### Prediction of Ag85A protein high-affinity peptide

Human HLA-A*0201 Ag85A high-affinity peptides (KLIANNTRV, GLPVEYLQV) were selected according to previous research [38]. Amino sequence of Ag85A protein was obtained from the website (https://www.ncbi.nlm.nih.gov/protein/). NetMHCpan (http://www.cbs.dtu.dk/services/NetMHCpan/) was used to predict mice H-2-D^b^, H2-IA^b^ high-affinity peptides. Finally, BCG Ag85A high-affinity peptides of mice H-2-D^b^ (SGGANSPAL) and mice H-2-IA^b^ (YHPQQFVYAGAMSGLLD) were selected (Table 1).

**Table 1:** Prediction of high-affinity peptides of C57BL/6J mice

Selected MHC-I sequence: SGGANSPAL			
Initial position	Sequence	Rank	MHC-I type
71	SGGANSPAL	0.04	H-2-D^b^
136	QTYKWETFL	0.19	H-2-K^b^
71	SGGANSPALYL	0.13	H-2-D^b^
179	AIYHPQQF	0.21	H-2-K^b^
199	SQAMGPTLI	0.21	H-2-D^b^
242	KLIANNTRV	0.21	H-2-D^b^

Selected MHC-II sequence: YHPQQFVYAGAMSGLLD			
Initial position	Sequence	Rank	MHC-II type
184	QQFVYAGAMSGLL	0.29	H2-IA^b^
183	PQQFVYAGAMSGLL	0.43	H2-IA^b^
184	QQFVYAGAMSGLLD	0.48	H2-IA^b^
182	HPQQFVYAGAMSGL	0.53	H2-IA^b^
183	PQQFVYAGAMSGL	0.37	H2-IA^b^
182	HPQQFVYAGAMSGLL	0.59	H2-IA^b^
181	YHPQQFVYAGAMSG	0.57	H2-IA^b^
183	PQQFVYAGAMSGLLD	0.62	H2-IA^b^

Smaller rank means stronger bind level

### Peptide dissolution

Peptides were synthesized and GLPVEYLQV was labeled with FITC fluorescence (ChinaPeptides Co., Ltd., China). The purity of the peptides was over 98%. 1mg peptide was dissolved in 50 µL DMSO, and then, 950 µL PBS was added to dissolve thoroughly. At this time, the formulated peptide concentration was 1 mg/mL. Peptide mixture1 equals 1mg KLIANNTRV and 1mg GLPVEYLQV dissolved in 1mL PBS. Peptide mixture2 equals 1mg SGGANSPAL and 1mg YHPQQFVYAGAMSGLLD dissolved in 1mL PBS.

### Tumor cell lines and culture

Human melanoma cell lines A375 (HLA-A*0201) (presented by Beijing Cancer Institute, China) were cultured in DMEM medium. Murine melanoma cell line B16F10 (purchased from Shanghai Institute of Biochemistry and Cell Biology, China) were cultured in Roswell Park Memorial Institute (RPMI) 1640 medium. All media were supplemented with 10% heat-inactivated fetal bovine serum (FBS), 100IU/mL penicillin, and 100µg/mL streptomycin (Invitrogen). Cells were incubated at 37°C with 5% CO_2_ in a humidified incubator.

### Flow Cytometry

Flow cytometry analysis was performed using the following antibodies (BD Bioscience): Anti-mouse CD3-PE/Cy7, Anti-mouse CD11c-PE/Cy7, Anti-mouse CD8-PE/Cy5, Anti-mouse CD44-PE, Anti-mouse CD62L-FITC, Anti-mouse PD-L1-PE, Anti-mouse CD326 PE/Dazzle, Anti-mouse CD4-PE/Dazzle, Anti-mouse CD80-PE, Anti-mouse CD86-PE/Dazzle, and Anti-mouse CD45 FITC. All samples were suspended in FACS buffer and stained with 1 µL antibodies for 30 min at 4°C in darks, then washed twice and resuspended in FACS buffer before analysis. For *γ*-IFN detection, Human Cytometric Bead Array (CBA) kit and mouse CBA kit (BD Bioscience, USA) were used. The antibody dilution concentration was 1:100. Samples were analyzed with BD Accuri C6 (BD Bioscience, USA) and CytoFLEX (Beckman, USA).

### Immunofluorescence confocal imaging

A375 melanoma cells were seeded into a 24-well plate which was placed with cell climbing slices at the concentration of 1.5 × 10^4^ cells/well. The next day, GLPVEYLQV-FITC was added and incubated at 37°C for 2 h, then washed with PBS 3 times and incubated for 4 h again. After the medium was removed, the cells were fixed with 4% paraformaldehyde (PFA) and processed with Dil (Beyotime, China) and DAPI (Beyotime, China) stain. The cells were imaged using the laser confocal microscope (Leica, German).

### Peptides incubated with PBMCs

Peripheral blood mononuclear cells (PBMCs) from healthy donors (HLA-A*0201) and tumor patients (HLA-A*0201) were obtained by Ficoll density centrifugation and suspended in AIM-V medium (Gibco, USA). 100 µL PBMCs were seeded into a 96-well plate at the concentration of 1 × 10^6^ cells/mL. The positive control group (PHA, 2.5 µL/100 µL), negative control group (A-IMV, none), and sample group (peptide mixture1, 2.5 µL/100 µL) were set, respectively. Each group set 3 wells. PBMCs were incubated with peptide mixture1 for 24 h at 37°C, and then, 40 µL supernatant was collected each well for detecting *γ*-IFN by flow cytometry. All experiments were run at least three times.

### Peptides incubated with tumor cells

A375 cells were cultured in the tubes at the concentration of 5 × 10^5^ cells/mL. GLPVEYLQV-FITC was added at different concentrations (0.5, 2.5, 5, 10, 20, 50 µg/mL) and co-incubated at 37°C for 2 h, then washed with PBS for 3 times to detect FITC fluorescence intensity on tumor cells by flow cytometry. In addition, 10 µg/mL GLPVEYLQV-FITC was added to tumor cells and FITC fluorescence intensity on tumor cells was detected at different time points (0, 0.5, 2, 3, 4, 5, 24, 48 h) by flow cytometry.

### Peptides incubated with tumor cells and PBMCs

The killing efficiency of HLA-A*0201 PBMCs was assessed by CFSE/PI labeling cytotoxicity assay. A375 cells were labeled with 4 μM CFSE (Abcam, UK) for 10 min at 37°C. Labeling was discontinued with the addition of a fivefold volume of RPMI 1640 supplemented with 10% FBS. CFSE-labeled tumor cells were then incubated with PBMCs (1 × 10^6^/mL) and peptide mixture1 (10 µg/mL) at various effector-to-target ratios (0:1 1:1 5:1 10:1 20:1) for 20 h. Tumor cell death was determined through the addition of PI (Sigma, USA). Samples were analyzed using flow cytometry.

### Construction and verification of BCG immunized mice

Female C57BL/6J mice (supplied by GemPharmatech Co. Ltd., Nanjing, China) weighing 12–16 g (4 weeks old) were housed under specific pathogen free (SPF) condition. Mice were immunized by intradermal injection of 0.1 mL BCG suspension (Chengdu institute of biological products, China) containing 3 × 10^5^ cfu at -Day28 and -Day21, respectively. At Day0, 0.1 mL of TB-PPD was injected intradermally into the abdominal skin of BCG immunized mice (Figs. 3a, 4a). These were then observed at 12, 24, and 48 h. Redness or induration > 5 mm at the injected site lasting for 48 h was defined as PPD-positive, indicating the successful construction of BCG immunized mice [1, 12].

### Induction of mature DC cells

Mouse bone marine-derived dendritic cells (BMDC) were taken and re-suspended in A-IMV medium with cell concentration of 5 × 10^5^/mL. BMDC suspension was added to a 24-well plate (5 × 10^5^/well), along with recombinant mouse GM-CSF (25ng/mL) and mouse IL-4 (10 ng/mL). Cultured in a constant temperature incubator, the medium was replaced in half doses at Day3 and Day6, respectively, and supplemented with recombinant mouse GM-CSF (25ng/mL) and mouse IL-4 (10 ng/mL). Mature DC cells can be obtained by collecting suspended cells on the eighth day.

### Peptides incubated with DC and T cells

The mature DC cells were then suspended into a 96-well plate (100 µL/well), and the cell content was 1 × 10^5^/well. The peptide mixture2 was added with 2 µL (10 µg/mL) per well. After 4 h, 1 µL cell stimulating factor R848 (3 µg/mL) was added. After another 30 min, 1 µL LPS (50 ng/mL) was added. T cells from mouse spleen and lymph nodes were suspended to 1 × 10^6^/mL with A-IMV containing 10% FBS, then added to 96-well plates (100 µL/well) and co-incubated for 18–24 h. 40 uL/well supernatant was collected for CBA detection of *γ*-IFN.

### Tumor outgrowth studies

C57BL/6J mice were injected subcutaneously with B16F10 cells (2 × 10^5^ suspended in 100 µL PBS) at Day0. The length and width were measured every other day and tumor volume was calculated following the formula: volume = length × width × width × 0.5. The mice were treated after the tumor volume reached 50 mm^3^. For the study compared the efficacy of peptide and BCG, BCG preimmunized mice (PPD-positive) were randomized into 3 groups (*n* = 6) and treated every four days with intratumor injection of 100 µL BCG (BCG-BCG), peptide mixture2 (BCG-PEPTIDE) or PBS (BCG-PBS). Mice not previously immunized with BCG were randomized into 2 groups (*n* = 6) and treated every four days with intratumor injection of 100 µL BCG (PBS-BCG) or peptide mixture2 (PBS-PEPTIDE). For another study combined with anti-PD1 antibody, PPD-positive mice were randomly divided into 4 groups including PBS, PEPTIDE, anti-PD1 and PEPTIDE + anti-PD1 (*n* = 5). Then, 100 µL peptide mixture2 (2 mg/mL) was injected intratumorally at day1 and 200 µL (1mg/mL) murine anti-PD-1 antibody (presented by TopAlliance Co., LTD, Suzhou, China) was injected intraperitoneally at day3 on a 4 day cycle (Figs. 3a, 4a).

### Side effects evaluation and tumor microenvironment analysis

Mice were weighed every two days and sacrificed by inhaled anesthesia followed by cervical dislocation either when the average tumor diameter exceeded 20 mm or when tumors became ulcerated. The tumor tissues, lymph nodes and spleens were excised and prepared as single cell suspensions for flow cytometry. For safety studies, 2 mice from each group were randomly selected and main organs (including heart, liver, spleen, lung and kidney) were harvested, fixed in 4% paraformaldehyde, sectioned, and stained with H&E.

### Statistical analysis

GraphPad Prism V.8.0 (GraphPad Software, San Diego, California, USA) was used for all statistical analyses. Data are presented as mean ± SEM. Student’s *t* test or one-way ANOVA test was used to determine the significance between groups. K-M survival curve was plotted and significance test was performed by Log-rank method. Statistical significance was confirmed when *p* < 0.05. ns, not significant; *p < 0.05; **p < 0.01; ***p < 0.001.
